# Lyophilization Based Isolation of Exosomes

**DOI:** 10.3390/ijms241310477

**Published:** 2023-06-22

**Authors:** Rida e Maria Qazi, Zahra Sajid, Chunqiu Zhao, Irfan Hussain, Fizza Iftikhar, Muhammad Jameel, Fawad Ur Rehman, Afsar Ali Mian

**Affiliations:** 1 Centre for Regenerative Medicine and Stem Cells Research, The Aga Khan University, 1st Flour, Juma Building, Stadium Road, Karachi 74800, Sindh, Pakistan; 2 State Key Lab of Bioelectronics, Southeast University, Sipailou 2, Nanjing 210096, China

**Keywords:** exosomes, nano biopolymers, lyophilization, drug delivery, Ph+ leukemia

## Abstract

Exosomes are nanoscale extracellular vesicles which regulate intercellular communication. They have great potential for application in nanomedicine. However, techniques for their isolation are limited by requirements for advanced instruments and costly reagents. In this study, we developed a lyophilization-based method for isolating exosomes from cultured cells. The isolated exosomes were characterized for protein content using Bradford assay, and for size distribution and shape using scanning electron microscopy (SEM) and nanoparticles tracking analysis (NTA). In addition, CD63, CD9, CD81, HSP70 and TSG101 were evaluated as essential exosomal surface markers using Western blot. Drug loading and release studies were performed to confirm their drug delivery properties using an in vitro model. Exosomes were also loaded with commercial dyes (Cy5, Eosin) for the evaluation of their drug delivery properties. All these characterizations confirmed successful exosome isolation with measurements of less than 150 nm, having a typical shape, and by expressing the known exosome surface protein markers. Finally, tyrosine kinase inhibitors (dasatinib and ponatinib) were loaded on the exosomes to evaluate their anticancer effects on leukemia cells (K562 and engineered Ba/F3-BCR-ABL) using MTT and Annexin-PI assays. The expression of MUC1 protein on the exosomes isolated from MCF-7 cells also indicated that their potential diagnostic properties were intact. In conclusion, we developed a new method for exosome isolation from cultured cells. These exosomes met all the essential requirements in terms of characterization, drug loading and release ability, and inhibition of proliferation and apoptosis induction in Ph+ leukemia cells. Based on these results, we are confident in presenting the lyophilization-based exosome isolation method as an alternative to traditional techniques for exosome isolation from cultured cells.

## 1. Introduction

Biomimetic nanotechnology has vastly impacted biomedicine by providing a robust scaffold for drugs, RNA interference (RNAi), protein delivery to desired tissues, and drug-free therapies. Recently, during the COVID-19 pandemic, nanotechnology assumed a prominent role in the development of mRNA vaccines and diagnostic kits worldwide [1,2,3]. Nevertheless, the translation of traditional nanomaterials to the bedside is challenged by adverse effects, poor target recognition and high costs [4,5]. In this regard, exosomes are a welcome option for addressing the aforementioned limitations. 

Exosomes are extracellular vesicles with a size range of 30–200 nm, secreted by almost all types of cells. They are involved in intercellular communication by having multiple payloads of DNA, RNA, RNAi, proteins, viruses, lipids, small molecules, and soluble factors [6,7]. These cargos are delivered from parent (donor) to recipient cells in a paracrine manner [8]. Exosomes have inherited payload-delivery ability. This cargo delivery ability makes them ideal candidates for drug delivery by loading the desired cargo either inside or on their surface [9,10]. Exosomes may also be employed as diagnostic markers for certain diseases, i.e., neurodegenerative diseases and cancers [10,11]. The exosomes also benefit from a natural ability to traverse all physiological barriers in the body, and, pertinently, the blood–brain barrier (BBB), which presents a significant obstacle in the amelioration of brain disease or resection of brain cancers [12]. Exosomes can be isolated from all body fluids, including blood plasma, urine, cerebrospinal fluids, amniotic fluids, pleural fluids, ascites, bronchoalveolar leverage, cell culture, etc. They are abundantly present in all body fluids with an average of 3 × 10^6^ particles/µL and a flotation density of 1.10–1.18 g/mL [13]. With such higher concentration and easy accessibility to all body organs and system, the exosome-based cell-free therapies present significant advantages in biomedicine [14].

There are several efficient methods for the isolation of exosomes, including prototype ultracentrifugation, which is used in 80% of extracellular-based research, density gradient reagents via poly-ethylene glycol (PEG), size exclusion chromatography (SEC) and immunoprecipitation, etc. [15]. However, these methods have certain limitations, i.e., the high cost of ultracentrifuges or commercial kits and their availability in low-income countries. In addition, the purity, size, and concentration of isolated exosome samples and their functionality depend on the isolation method employed [16]. Chen et al. evaluated different methods for extracting exosomes from synovial tissue: ultracentrifugation, SEC and PEG (8%). They found that the exosomes isolated through the PEG-based technique could express calnexin as a sign of contamination with other than exosomal proteins [17]. Similarly, the exosome payload could be influenced by the isolation method used [18]. 

The Philadelphia chromosome (Ph) is the der(22) of the reciprocal translocation t(9;22)(q34;q11), which encodes the BCR-ABL, the oncogenic driver of chronic myeloid leukemia (CML) and 20–25% of cases of adult acute lymphatic leukemia (ALL) [19,20]. Tyrosine kinase inhibitors (TKIs) which target BCR-ABL, such as imatinib, dasatinib and ponatinib, have resulted in durable cytogenetic and molecular remissions in most CML patients and increased remission rates in Ph+ ALL patients [21]. However, survival is impaired by a relapse after the initial response [22]. The relapse is due to drug resistance which has multiple mechanisms, including drug availability in the target leukemic cells [23].

**Figure 1:**
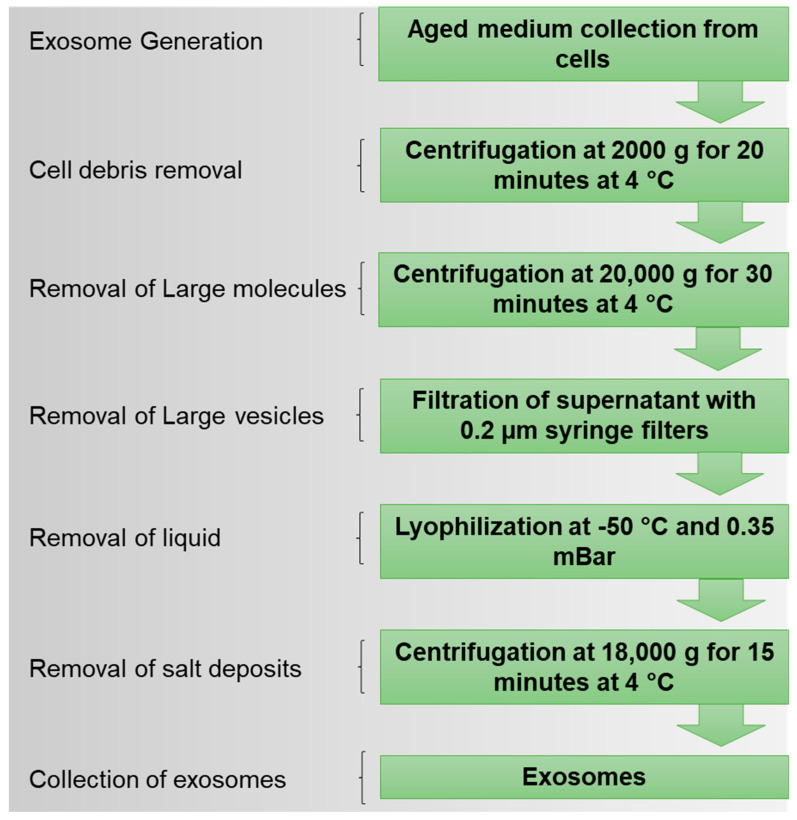
Design of lyophilization method for isolation of exosomes from cell culture media.

In this study, we used the lyophilization (freeze-drying) technique to establish a method for the isolation of exosomes, a subclass of extracellular vesicles (Figure 1). Unlike ultracentrifuge and other reagent-based methods, lyophilization is cost-effective and straightforward. We further employed these lyophilized exosomes to deliver tyrosine kinase inhibitors (TKIs) to the Ph+ leukemia established cell line (K562) and BCR-ABL-engineered murine cells (Ba/F3-P185). These exosomes could help to deliver the TKIs to the target leukemia cells and induce apoptosis.

## 2. Results

### 2.1. Isolation of Exosomes

Exosomes were isolated using the lyophilization method and the results were compared with a commercial kit (total exosome isolation, cell culture). It was found that the lyophilization method was not inferior to the kit method. Lyophilization could efficiently isolate exosomes from suspension (leukemia) and adherent (breast cancer) cultured cells.

### 2.2. Exosome Characterization

**Figure 2:**
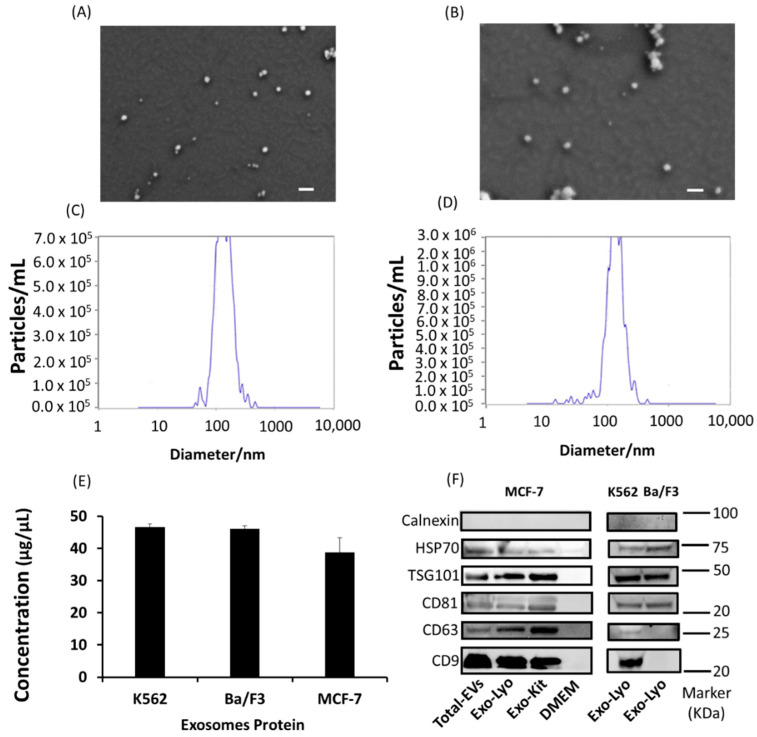
Characterization of exosomes isolated using lyophilization method from suspension and adherent cell lines. (**A**) K562 and (**B**) MCF-7 scanning electron micrograph for exosome size and shape. Scalebar: 200 nm. NTA analysis of exosomes isolated from (**C**) K562 and (**D**) MCF-7 cells for size distribution (nm) and number of particles per mL. (**E**) Concentration of exosomes isolated from K562, Ba/F3, and MCF-7 cells. (**F**) Characteristic Western blot markers of total extracellular vesicles (EVs) and exosomes (Exo) isolated via the lyophilization (Lyo) method and the commercial exosomes isolation reagent (total exosomes isolation, cell culture) from MCF-7, K562, and Ba/F3 cell lines. (N = 3).

Initially, the exosomes isolated from adherent (MCF-7) and suspension (K562) cell lines via the lyophilization method were characterized using scanning electron microscopy (SEM), which revealed evenly distributed, round-shaped extracellular vesicles that were in the exosome size range (<200 nm) (Figure 2A,B and Figure S1). Nanoparticle tracking analysis (NTA) was performed to obtain the percentage distribution of exosomes on the bases of diameters of 130.9 (60%) and 174.2 nm (34%) with a mean size of 138.4 ± 47.8 nm in the K562 cells (Figure 2C), and 125.7 ± 47.8 nm (97.4%) with a mean size of 144.3 ± 52.3 nm in the MCF-7 cells (Figure 2D). The exosome protein levels were quantified using Bradford assay and found to be 46.60 ± 0.9833, 49.04 ± 0.9673, and 38.75 ± 4.57 µg as total protein for K562, Ba/F3, and MCF-7 cells, respectively (Figure 2E). Finally, we used Western blot to evaluate the exosomes for their essential signature surface protein, including HSP70, TSG101, CD63, CD81, CD9, and Calnexin. Both adherent and suspension cell lines significantly expressed signature surface proteins pertinent for exosome characterization. The primary antibodies of CD63 and CD9 were specific for humans, since Ba/F3 is mouse cell line, therefore we could not detect these markers in Ba/F3 isolated exosomes. A comparison of lyophilization-isolated exosomes with those obtained using the commercially available kit (total exosomes isolation, cell culture) revealed no significant difference (Figure 2). 

### 2.3. Drug Loading and Release

**Figure 3:**
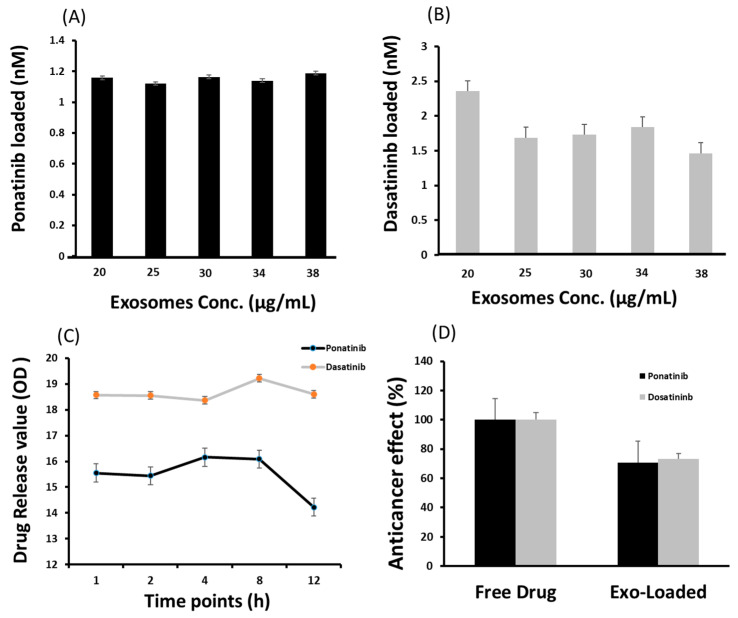
Cargo loading and release ability of exosomes isolated using lyophilization method. (**A**) is ponatinib and (**B**) is dasatinib (tyrosine kinase inhibitors) loaded in nM on various concentrations of exosomes in µg/mL. (**C**) is the drug release under standard incubation conditions (37 °C, pH 7.4) and shear force (10 g) for 12 h. The optical density (OD) value for ponatinib and dasatinib was recorded at 285 and 233 nm, respectively. (**D**) shows the relative drug delivery ability to target cells, evaluated by the anticancer effect normalized with a free drug effect. (N = 3).

Ponatinib (2 nM) and dasatinib (5 nM) were selected for drug-loading and release studies on exosomes. Exosomes with various concertations of 20, 25, 30, 34, and 38 µg/mL proteins were loaded with drugs. There was no significant difference in the drug loading ability of the various exosome concentrations, i.e., in 20 µg and 38 µg exosomes 1.18 nM and 1.58 nM of ponatinib was loaded, respectively (Figure 3A). However, the highest loading concentration of dasatinib (2.35 nM) was observed in 20 µg exosomes (Figure 3B). In vitro drug release studies have suggested that dasatinib release reaches its maximum (19.22 optical density (OD)) after 8 h and then declines. In ponatinib-loaded exosomes, a slight decrease was observed in the drug concentration at the 8 h timepoint compared to 4 h (16.09 vs. 16.16, respectively). However, a sharp decline in drug concentration was observed at the 12 h time point, i.e., 18.61 and 14.22 for dasatinib and ponatinib, respectively (Figure 3C). Drug delivery capability was also confirmed by the anticancer effect of an exosomes-loaded drug vs. a free drug. We observed a 70% relative anticancer effect of the exosomes-loaded TKIs, suggesting a therapeutically active drug concentration (Figure 3D).

### 2.4. Cargo Delivery to Target Cells

**Figure 4:**
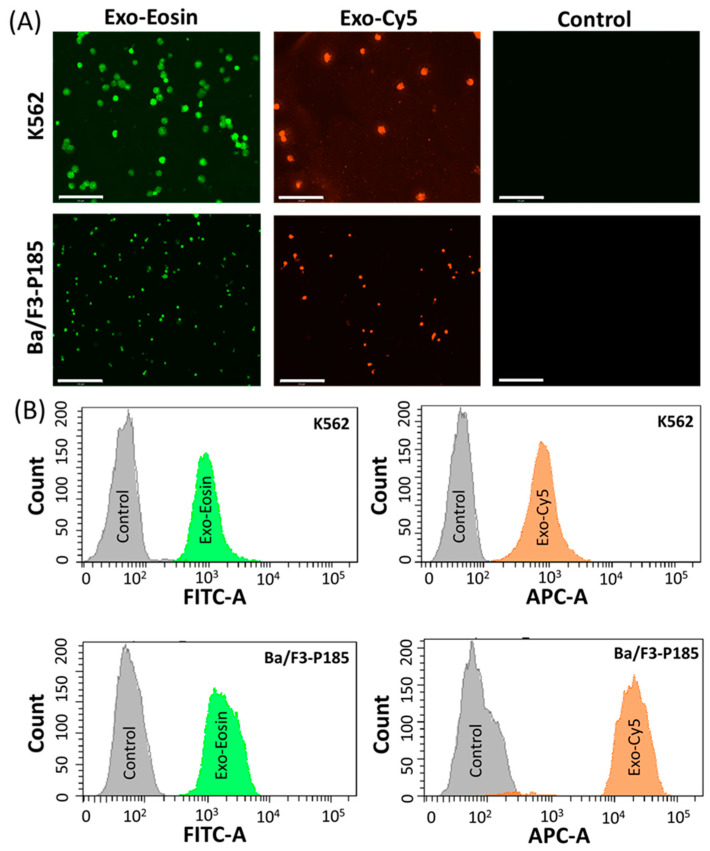
Cargo delivery ability of exosomes in targeted K562 and Ba/F3-P185 cells. (**A**) shows the fluorescence imaging of K562 and Ba/F3-P185 cells treated with eosin-loaded exosomes (Exo-Eosin) and Cy5-loaded exosomes (Exo-Cy5). The excitation and emission wavelength of eosin are 525 and 546 nm, respectively. The excitation and emission wavelength of Cy5 are 651 and 670 nm, respectively. (**B**) shows the flow cytometry data for uptake of Exo-Eosin and Exo-Cy5 in K562 and Ba/F3-P185 cells. Controls are the untreated cells.

The exosomes were isolated from the target cells, i.e., K562 and Ba-/F3-P185, and then loaded separately with eosin and Cy5 dyes. Fluorescence imaging demonstrated that exosomes could efficiently deliver eosin and Cy5 to target cells (Figure 4A). In addition, the flow cytometry data were consistent with the fluorescence imaging (Figure 4B). 

### 2.5. Antileukemia Effect

#### 2.5.1. Cell Proliferation

**Figure 5:**
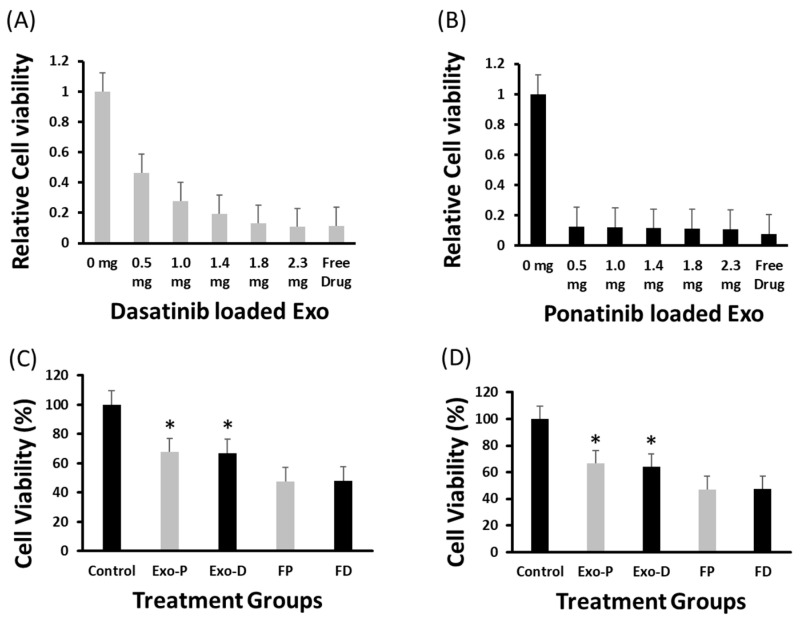
Antileukemia effect of drug-loaded exosomes. The MTT assay-based relative cell viability of dasatinib- and ponatinib-loaded exosomes and their antileukemia effect against K562 cells at various exosome concentrations are shown in (**A**,**B**), respectively. The probability value was highly significant (<0.001) after 72 h of incubation. (**C**,**D**) show the viability of K562 and Ba/F3-P185 cells after 48 h of incubation, respectively. Controls are the naïve exosomes treated cells. Exo-P, exosomes with ponatinib; Exo-D, exosomes with ponatinib; FP, free ponatinib; and FD, free dasatinib. * is the *p* < 0.05. (N = 3).

MTT assay revealed that the dasatinib anticell proliferation effect was directly proportional to the exosome concentration, i.e., 0.5 mg exosomes could induce a 54% anticancer effect, whereas 2.3 mg exosomes resulted in 89.2% growth inhibition that was equal to the effect (88.7%) of a standard free drug (5 nM) (Figure 5A). For dasatinib, cell viability significantly decreased with an increase in drug-loaded exosomes. Similarly, ponatinib-loaded exosomes at a lower concentration, i.e., 0.5 mg, could induce an 87.4% anticell proliferation effect and remained almost identical at all concentrations, even for the maximum 2.3 mg concentration of 89.4%. The free ponatinib could inhibit cell proliferation by 92.2% (Figure 5B). By utilizing 20 µg exosomes for dasatinib and ponatinib drug delivery, we observed ~67% cell viability compared to free drugs, which was <50% for both K562 and Ba/F3-P185 cell lines (Figure 5C,D). The cell count for K562 and Ba/F3-P185 cells after treatment with dasatinib and ponatinib-loaded exosomes is provided in Figure S4.

#### 2.5.2. Apoptosis

**Figure 6:**
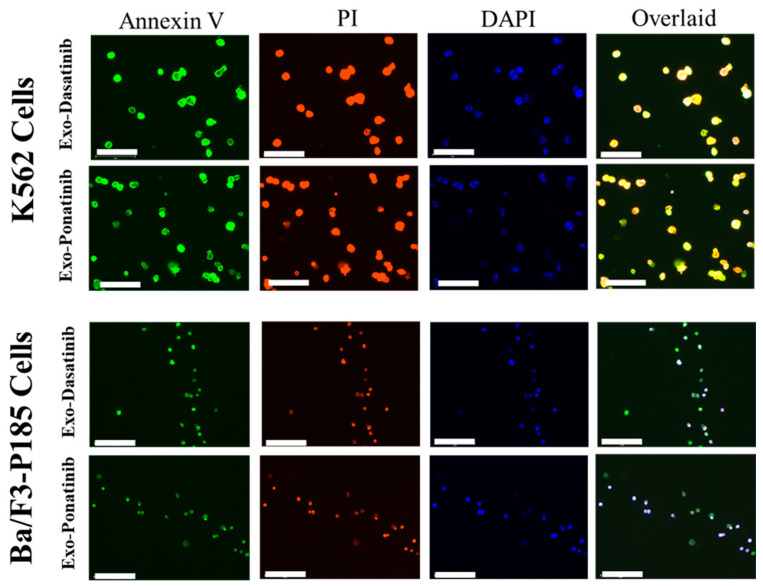
Fluorescence images of apoptosis induced in leukemia cells after treatment with drug-loaded exosomes. The excitation and emission wavelengths of Annexin-V are 485 and 535 nm, and for PI they are 560 and 595 nm, respectively. DAPI was used to stain the cell nucleus. The scale bar is 75 µm.

**Figure 7:**
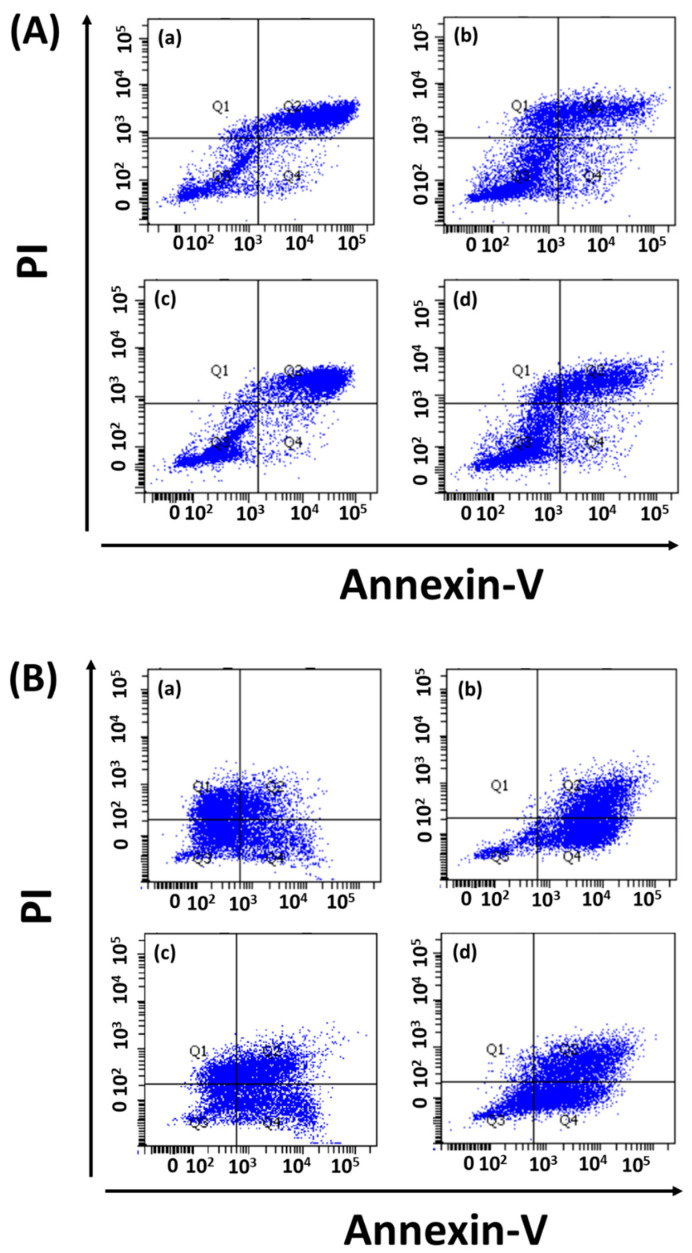
Apoptotic effect of drug-loaded exosomes on leukemia cells. Flow cytometry data for (**A**) K562 cells and (**B**) Ba/F3-P185 cells treated with exosomes loaded with (**a**) dasatinib and (**b**) ponatinib, in comparison with (**c**) free dasatinib and (**d**) free ponatinib. (**a**–**d**) remains same for both (**A**,**B**).

Drug-loaded exosome apoptosis induction was evaluated using Annexin-PI assay. FITC detected Annexin-V signals for early apoptosis, and the Cy5 channel could detect PI for late apoptosis during fluorescence microscopy. In flow cytometry experiments, Annexin-V and PI were detected by the FITC and PE channels, respectively. Fluorescence microscopy revealed equal signal strengths for exosome-loaded drugs and free drugs (Figure 6, Figures S2 and S3). In addition, K562 cells treated with drug-loaded exosomes (i.e., dasatinib, ponatinib) showed 61% apoptosis compared to treatment with free drugs (~40%). Similarly, dasatinib-loaded exosomes could induce 37% apoptosis in Ba/F3-P185 cells compared to 92.3% with free drug treatment, as shown in Figure 7. Ponatinib-loaded exosomes induced 65.8% apoptosis in Ba/F3-P185 cells compared to 78.9% with free drug treatment. Interestingly, treatment with dasatinib- and ponatinib-loaded exosomes could induce necrosis in Ba/F3-P185 cells of 29.4% and 14.9%, respectively. No effect was observed in untreated controls, as shown in Figure S5.

## 3. Discussion

The ultracentrifuge and reagent-based protocols for exosome isolation are well-established and have higher efficiency. However, these methods are limited by issues relating to costly reagents, higher freight charges, and prolonged supply times because of frequent lockdowns during the COVID-19 pandemic. Furthermore, in addition to the affordability issue, national and international policies regulate the use of ultracentrifuges because of their potential applications in the enrichment of radioactive materials. Therefore, our study aimed to find an equally efficient alternative to the use of ultracentrifuges or reagents for the isolation of exosomes from cultured cells. 

Lyophilization (freeze-drying) is now commonly used for the prolonged storage of enriched exosomes. However, to the best of our knowledge, its application in exosome isolation is novel. This method is simple and comprises the same differential centrifugation steps used for exosome isolation, with the exception of ultracentrifugation [24]. Interestingly, most exosome storage buffers have a recipe for maintaining their structure and functionality. For instance, 4% trehalose or 10% *w*/*v* sorbitol and sucrose are favored for freeze-drying of enriched exosomes pellet. Similarly, the ideal pH is 6.5–7.5, and amino acids, glutamic acid and supplementation of 1% FBS are favored to avoid cryoshock and maintain the structural integrity of the exosome pellet [25,26]. Likewise, for cell culture media, Dulbecco’s Modified Eagle Medium (DMEM) is rich in glucose, having a pH of 6.8–7.4, primarily rich in L glutamate and supplemented with 10% FBS [27]. Analogous to lyophilization-based storage media for exosome pellets, the cell culture media can protect the exosomes. Therefore, the lyophilization-based technique is able to isolate the exosomes by freeze-drying the aged cell culture medium. However, we still need to rely on traditional exosome isolation techniques for washing. Indeed, no isolation method can completely purify the exosome sample from protein contamination and render it inert to exosomal cargo, although SEC or ultracentrifugation can partially meet the challenge [28].

A comparative study of the exosome isolation technique evaluated the efficiency, purity and reproducibility of various commercial kits for exosome purification and their miRNA quality in plasma and serum samples. The serum was found suitable for exosome isolation and had a greater variety of exosomal miRNA. It was also found that every isolation method exhibited certain limitations regarding purity, exosomal number and cargo content [29]. In contrast, Tang et al. reported that plasma was more feasible for exosome isolation [30]. No single method is equally efficient for all types of exosome samples; even ultracentrifugation is limited by lower yield compared to commercial kits, but with relatively higher purity. Similarly, Rekker et al. compared ultracentrifugation with commercial kits and found that the isolation method could influence the miRNA payload on exosomes [18], and the same is true for exosomal cytokines that can be used for the diagnosis of certain diseases [31]. These studies suggest that exosome cargo detection and isolation wholly depend upon the isolation procedure adopted. The lyophilization-based exosome isolation method is a valid option by virtue of its simplicity and cost-effectiveness.

In our study, the exosomes were isolated using the lyophilization method and characterized for size and shape using scanning electron microscopy (SEM). In addition, NTA was used to accurately reveal the concentration and size of the exosomes within a sample. The average size of the extracellular vesicles was around 140 nm for both the adherent and suspension cell lines, which is in the range of the reported size of exosomes (30–200 nm) [32,33]. The average concentration of exosomes based on the protein was also satisfactory for downstream applications. The exosome surface protein markers are considered standard for their characterization. For instance, CD9, CD63, and CD81 are endosome-specific tetraspanins. CD9 was first identified on exosomes isolated from dendritic cells [34]. TSG101 and HSP70 are components of the endosomal sorting complexes required for the transport (ESCRT) complex, and have an essential role in exosome generation [35]. Calnexin is an endoplasmic reticulum marker and is mainly evaluated as a negative control for exosomes. However, Saludas et al. reported its presence on relatively large vesicles, whereas CD63 was on small ones [36]. Western blot testing confirmed that all these important markers, with the exception of calnexin, were present on the surface of the isolated exosomes.

We then evaluated these isolated exosomes for drug loading and release ability by loading them with dasatinib and ponatinib, which are used as tyrosine kinase inhibitors for Ph+ leukemia. It was observed that the exosomes could efficiently load and deliver the drug to target cells. Drug release studies were performed in vitro by collecting samples at various time points. At 8 h, the drug release was optimum, whereas a decline was observed at 12 h. In addition to antileukemic drug delivery, commercial dyes (Cy5 and eosin) were also successfully loaded on and delivered by exosomes to target K562 and Ba/F3-P185 cells. Previously, Wei et al. demonstrated that the release of the drug doxorubicin by exosomes in the tumor microenvironment (osteosarcoma) was triggered by lower pH (<6.5) [37]. This is confirmed by our results, because K562 is a leukemia cell line and has a lower pH which triggers the release of the drug from the exosomes.

Exosome-based specific cell targeting can be achieved actively by surface modification through targeting ligands [38,39] or passively via parent cell affinity [40,41]. In this study, we chose exosome cell affinity-based drug delivery by employing K562 and Ba/F3-P185 isolated exosomes for parent cell treatment [42]. The antileukemic effect was evaluated according to cell proliferation and apoptosis induction in target cells after treatment with dasatinib- and ponatinib-loaded exosomes. A nonsignificant difference was observed between the various concentrations of ponatinib-loaded exosomes, suggesting that a lower concentration of exosomes is sufficient to lower cell viability. Apoptosis data suggested that exosomes loaded with dasatinib and ponatinib could induce around 40% higher apoptosis in K562 cells. However, this was not the case for BCR-ABL-engineered murine cells (Ba/F3-P185), i.e., 20% lower apoptosis was found in drug-loaded exosomes compared to free drugs. It is well known that the response of K562 to TKIs is better than that of Ba/F3-P185 [43].

Exosome application in nanomedicine is favored for drug delivery and diagnosis [44]. In drug delivery, we could successfully deliver cell imaging dyes (i.e., Cy5 and eosin) and antileukemia drugs (i.e., dasatinib and ponatinib). For the diagnostic merit of this new method, exosome surface markers, other than pivotal characterization markers, were also evaluated. MCF-7 cells have a high expression of membrane mucin-1 (MUC1) protein [45]. These proteins are also used as exosome markers for breast cancer [46,47]. Therefore, we checked MUC1 expression on the exosomes isolated from MCF-7 cells (Figure S6). We could successfully detect the MUC1 expression on the exosome lysates, and this vouches for the inertness of the lyophilization-based exosome isolation technique.

In conclusion, we have developed a novel method to isolate extracellular vesicles (exosomes) from adherent and suspension cell cultures. The isolated extracellular vesicles had typical exosome structures confirmed by SEM and NTA and could deliver the drug and dye cargo to targeted cells. The antileukemia effect achieved by reducing cell proliferation and inducing apoptosis was evaluated in the human Ph+ positive leukemia cell line (K562) and murine-engineered myeloid cells (Ba/F3-P185). The presence of MUC1 on the MCF-7 isolated exosome surface provided additional evidence of the integrity of surface protein after isolation by lyophilization. This novel modality provides an alternative to traditional exosome isolation techniques with potential application in nanomedicine (drug delivery and diagnosis).

## 4. Materials and Methods

### 4.1. Materials

All cell culture media (DMEM, RPMI 1640) and cell culture supplements were purchased from Gibco Inc. (Billings, MT, USA), and cell culture plasticware was obtained from ThermoFisher, unless otherwise mentioned. Millipore deionized water of 18.0 Ω/cm^2^ was used for reagent dilution when required. MTT assay reagents (Cell Proliferation Kit I (MTT) Cat# 11465007001) were purchased from Roche. Exosome surface marker antibodies were obtained from Abcam (Cat# ab275018: Exosome Panel; CD9, CD63, CD81, TSG101, Hsp70, Calnexin). Annexin-V was purchased from BD Bioscience (Cat# 556419), and propidium iodide (PI) was obtained from Invitrogen, ThermoFisher Scientific (Waltham, MA, USA). The exosome isolation kit (total exosome isolation (cell culture), Cat# 4478359) was purchased from Invitrogen^TM^ by Thermo Fisher Scientific.

### 4.2. Cell Culture

Breast cancer cell lines (MCF-7), Philadelphia (+) Myeloid Leukemia cell lines (K562), and murine pro-B cell lines (Ba/F3) were obtained from the German Collection of Microorganisms and Cell Culture (DSMZ, Braunschweig, Germany). All suspension cell (K562 and Ba/F3) cultures were maintained in the RPMI-1640 medium, whereas MCF-7 cells were cultured in DMEM. Both media were supplemented with 10% (*v*/*v*) fetal bovine serum (FBS), 1% glutamate, and 1% streptomycin–penicillin solution (*v*/*v*). Ba/F3 cells were maintained in 10 ng/mL mIL-3 (Cell Concepts, Umkirch, Germany) supplemented medium until modified with BCR/ABL, i.e., P185. MCF-7 was trypsinized with 0.025 trypsin-EDTA when the confluence exceeded 90%.

### 4.3. Retroviral Infection

PINCO vectors were used to transfect the ecotropic Phoenix packaging cells, as mentioned earlier [48]. The retroviral supernatant was collected after 36 h. Ba/F3 cells were cultured in retronectin-coated nontissue-culture 24-well plates and maintained in a medium supplemented with mIL-3. These cells were then exposed to the collected retro-viral supernatant for three hours at 37 °C. The survival of the Ba/F3 cells without mIL-3 supplemented medium confirmed successful (BCR-ABL) P185 transduction in the target cells (Ba/F3).

### 4.4. Lyophilization for Exosome Isolation

All cells in the log phase with 90% confluence were incubated in culture media for 72 h, and then the aged cell culture media were collected for exosome isolation. Collected aged media were initially centrifuged at 2000× *g* for 20 min at 4 °C. The supernatant was then collected and centrifuged at 20,000× *g* for 30 min at 4 °C. Again, the supernatant was collected and filtered through a 0.20 µm PES syringe filter (Thermoscientific, Waltham, MA, USA). The filtered medium was kept at −80 °C until the medium was completely frozen (~4–12 h). The samples were then placed in a lyophilizer (Labconco, Freeze Zone 2.5 C) under −50 °C and 0.35 mbar vacuum pressure for 24–36 h. The duration of complete lyophilization of the sample depends on the sample volume. In this experiment, we used a 30 mL conditioned medium. After lyophilization, the freeze-dried samples were reconstituted in 2.0 mL PBS and then centrifuged at 18,000× *g* for 15 min. The residual salts were pelleted out from the samples, and a supernatant containing exosomes was collected. The exosome-enriched supernatant was then used for downstream experiments and characterization.

### 4.5. Exosome Characterization

The following methods were used for exosome characterization.

#### 4.5.1. Quantification

Exosomes were quantified on protein content bases using the Bradford standard protein assay. We diluted 2.0 µL of the samples in 38 µL of deionized distilled water to obtain a final volume of 40 µL in a 96-well plate. Then, 10 µL of the dilute samples were placed in each well of 96-well plates, and 190 mL of 1 × Bradford assay reagent was added (Quickstart, Bradford Protein Assay Kit 1, BioRad, Hercules, CA, USA) and incubated for 5 min. Next, the OD value was recorded using a microplate reader (Multiscan Sky, Thermoscientific) at 595 nm wavelength. Bovine serum albumin (Sigma-Aldrich, St. Louis, MO, USA) 50 µg/µL was run as the standard control.

#### 4.5.2. Electron Microscopy

The exosomes isolated using the lyophilization method were fixed in 10% formalin solution (*v*/*v*) for 10 min and washed with PBS (3×). Then, a 10 µL drop of PBS containing exosomes was placed on the silicon wafers and dried by overnight incubation. After gold sputtering, the samples were analyzed under a field-emission scanning electron microscope (Zeiss, ultra plus, Juelich, Germany) for determination of size and shape.

#### 4.5.3. Nanoparticle Tracking Analysis (NTA)

NTA was performed using ZetaView^®^ (particle matrix). The exosome samples were diluted in PBS (1:3) and then analyzed for concentration and size by capturing video for 0.5 s in 11 positions at room temperature. The maximum and minimum sizes were 1000 and 10 nm, respectively, and a 520 nm laser was chosen with minimum brightness set at 30. The data were then analyzed using ZetaView software version 8.04.12.

#### 4.5.4. Western Blot

Western blotting was performed as described in Sambrook’s protocols [49]. Briefly, exosomes were treated with radioimmunoprecipitation assay (RIPA) lysis buffer at a ratio of 1:2 and incubated at 4 °C for one hour. The lysates were centrifuged at 18,000× *g* for 15 min at 4 °C, and the protein supernatant was collected. Bradford assay was performed for protein quantification, as mentioned earlier. The proteins were denatured using Laemmli buffer at 95 °C for 10 min. The sample was loaded on 10% gel in a running buffer at 120 V (BioRad system). The protein-containing gels were then wet transferred onto a nitrocellulose membrane at 350 mA for 100 min. The membranes were blocked with 5% skimmed milk in PBST (*w*/*v*) (0.1% Tween-20 in PBS (*v*/*v*)). An Abcam exosome antibodies panel containing CD9, CD63, CD81, TSG101, Hsp70, and Calnexin was used for the primary antibodies (Cat# ab275018: Exosome Panel), whereas secondary antihuman mouse antibodies were used at 1:5000 dilution. For MUC1 expression, the blots were incubated in primary MUC1 antibody (MUC1 VU4H5) at a dilution of 1:500. All antibodies were diluted in 2% skimmed milk in PBST (*w*/*v*). After membrane blocking, samples were washed three times with PBST before every step. After incubation with ECL for 2.0 min in the dark at room temperature, the membranes were imaged using the BioRad Chemidoc imaging system.

#### 4.5.5. Drug Loading and Release Studies

For drug loading and release studies, we adopted a previously published method [38]. Briefly, dasatinib (5 nM) and ponatinib (2 nM) were selected as standard drugs for loading on exosomes at concentrations of 20, 25, 30, 34 and 38 µg/mL. Both drugs (dasatinib and ponatinib) were loaded separately on exosomes through the sonication method (3 cycles of 30-s pulses with 10 s of ice rest at 40% sonication power) using a probe Sonicator (Branson 150). The exosomes were rested on ice for 30 min and then incubated at 37 °C for one hour. The exosomes were then purified using a commercial kit (total exosomes isolation (cell culture), Cat# 4478359) at a 1:2 ratio and incubated overnight at 4 °C according to the manufacturer’s guidelines. They were finally pelleted out using centrifugation at 10,000× *g* for 1 h at 4 °C.

The drug-loaded exosomes were lysed using 1% triton-X solution (*v*/*v*) and incubated overnight at room temperature in the dark. They were then centrifuged at 18,000× *g* for 15 min. The supernatant was collected, and the optical density (OD) value was calculated using a spectrophotometer (DU^®^ 730, Beckman Coulter, Brea, CA, USA) at wavelengths of 233 nm and 285 nm for dasatinib and ponatinib, respectively. The OD values of the drug-loaded exosome supernatants were compared with those of free drugs.

For the evaluation of the drug release ability of the exosomes, dasatinib (5 nM) and ponatinib (2 nM) were loaded on exosomes (20 µg/mL) using the sonication method, as mentioned earlier. The exosomes were then resuspended in PBS (pH 7.4) and incubated in an automatic shaker at 37 °C with a shear force of 10 g. At time points of 1, 2, 4, 8 and 12 h, the samples were collected, and exosomes were pelleted out and supernatant was collected. The OD values of both drugs were measured and evaluated from supernatant.

#### 4.5.6. Cell Counting

Cell counting was performed using the Countess II (Invitrogen) cell counting system. Initially, a 10 µL sample was diluted in 40 µL of Trypan blue solution (Gibco) and loaded on the specialized disposable slide for cell Countess II. The number of live and dead cells and their percentages were determined.

#### 4.5.7. MTT Assays

K562 cells were cultured in 96-well plates with 10,000 cells per well in 50 µL volume. Exosomes isolated from K562 cells were preloaded with dasatinib (5 nM) and ponatinib (2 nM) and then added to each well at 0, 0.5, 1.0, 1.4, 1.8, and 2.3 mg so that the final total volume in each well was 100 µL. The free drug was kept as a positive control with a concentration of 5 nM dasatinib and 2 nM ponatinib. The samples were then incubated for 72 h under standard tissue culture incubation conditions. Similarly, Ba/F3-P185 and K562 cells were treated with 0.5 mg exosomes loaded with ponatinib (2 nM) and dasatinib (5 nM) and the same concentration of the free drugs for 48 h. Controls are the naïve exosomes treated cells. Next, 10 µL of MTT solution was added to each well and incubated for 4 h. Later, 100 µL of reagent 2 was added to each well and incubated overnight. OD values were read using a microplate reader (Multiscan Sky, Thermoscientific) at 575 nm wavelength.

### 4.6. Apoptosis Assays

#### 4.6.1. Flow Cytometry

K562 and Ba/F3-P185 cells were cultured in 24-well plates with 1 × 10^6^ cells per well. One group of cells was treated with exosomes loaded with dasatinib (5 nM) and ponatinib (2 nM), whereas the other group was treated with free dasatinib and ponatinib at the same concentration that was used for loading on exosomes (5 and 2 nM, respectively) as a positive control. Untreated cells were kept as a negative control. After 72 h of incubation, propidium iodide (PI) and Annexin-V were added to each well and incubated for 10 min. The cells were washed with PBS and resuspended in a resuspension buffer for flow cytometry using a BD FACSAria III. Propidium iodide has excitation (535 nm) and emission (595 nm) wavelengths and was detected using the PE channel. Similarly, Annexin-V has excitation (485 nm) and emission (535 nm) wavelengths, and its fluorescence was detected using the FITC channel. The data were analyzed using FACSDiva Version 6.1.3.

#### 4.6.2. Bioimaging

Cells were cultured and treated for bioimaging as described in the flow cytometry section. The cells were collected and fixed with 10% formalin (*v*/*v*) for 10 min. They were again incubated with DAPI solution (20 µM) for 20 min at room temperature. Because the K562 and Ba/F3-P185 cells are suspension cells, we used cytospin technology to fix the cells on glass slides by centrifugation at 50× *g* for 5 min at room temperature (Cytospin 4, Thermoscientific). The cells were then imaged using the EVOS microscopy system (EVOS, FL Auto 2 imaging system, Invitrogen, Waltham, MA, USA), using DAPI, FITC, and Cy5 channels.

Eosin was loaded on the exosomes isolated from the target cells, i.e., the K562 and Ba/F3-P185 cells. As mentioned earlier, 50 µL eosin was loaded on 20 µg exosomes using the sonication method. Then, the cells were ice-rested for 30 min and incubated at 37 °C for one hour. The commercial kit reagent (total exosomes isolation (cell culture), Cat# 4478359) was added at the ratio of 1:2 (kit reagent: exosomes with eosin) to remove unloaded eosin. After overnight incubation, the samples were centrifuged at 18,000× *g* for 15 min at 4 °C. The pellet was resuspended in PBS and inoculated with K562 and Ba/F3-P185 cells for 12 h. The treated cells were washed with PBS and fixed with 10% formalin, and cytospin was used for fixation on a glass slide. The cells were finally imaged using the FITC channel of the EVOS imaging system. Eosin excitation and emission are 525 and 546 nm, respectively.

### 4.7. Statistical Analysis

Data were initially recorded in MS Excel, and statistical software SPSS version 18 (IBM, Chicago, IL, USA) was used for results analysis using the Student’s *t*-test. A probability value of <0.05 was considered significant. All experiments were run in triplicate, and data are presented as the mean ± standard deviation (SD).

## Data Availability

Data is available upon request.

## Supplementary Materials

The following supporting information can be downloaded at: https://www.mdpi.com/article/10.3390/ijms241310477/s1.

## References

[1] Rai M., Bonde S., Yadav A., Plekhanova Y., Reshetilov A., Gupta I., Golińska P., Pandit R., Ingle A.P. (2022). Nanotechnology-based promising strategies for the management of COVID-19: Current development and constraints. Expert Rev. Anti-Infect. Ther..

[2] Vahedifard F., Chakravarthy K. (2021). Nanomedicine for COVID-19: The role of nanotechnology in the treatment and diagnosis of COVID-19. Emerg. Mater..

[3] Zhao C., Rehman F.U., Shaikh S., Qazi R.M., Sajid Z., Mian A.A., He N. (2023). Metallic nanoscale-knife application in cancer theranostics. Smart Mater. Med..

[4] Rehman F.U. (2018). Nanomedicine: Why it still taking long from “bench to bedside”. Biomed. Lett..

[5] Younas M.U., Hussain M., Akram A., Rasool S., Andleeb H., Ahmad S., Sharif M.S., Khan S. (2022). Toxicological assessment of nanoparticles and microplastics. Biomed. Lett..

[6] Syn N.L., Wang L., Chow E.K.-H., Lim C.T., Goh B.-C. (2017). Exosomes in cancer nanomedicine and immunotherapy: Prospects and challenges. Trends Biotechnol..

[7] Yáñez-Mó M., Siljander P.R.-M., Andreu Z., Bedina Zavec A., Borràs F.E., Buzas E.I., Buzas K., Casal E., Cappello F., Carvalho J. (2015). Biological properties of extracellular vesicles and their physiological functions. J. Extracell. Vesicles.

[8] Rehman F.U., Du T., Shaikh S., Jiang X., Chen Y., Li X., Yi H., Hui J., Chen B., Selke M. (2018). Nano in nano: Biosynthesized gold and iron nanoclusters cargo neoplastic exosomes for cancer status biomarking. Nanomed. Nanotechnol. Biol. Med..

[9] Kanninen K.M., Bister N., Koistinaho J., Malm T. (2016). Exosomes as new diagnostic tools in CNS diseases. Biochim. Biophys. Acta (BBA)-Mol. Basis Dis..

[10] Fu Y., Jiang C., Tofaris G.K., Davis J.J. (2020). Facile impedimetric analysis of neuronal exosome markers in Parkinson’s disease diagnostics. Anal. Chem..

[11] Shaikh S., Rehman F.U., Du T., Jiang H., Yin L., Wang X., Chai R. (2018). Real-time multimodal bioimaging of cancer cells and exosomes through biosynthesized iridium and iron nanoclusters. ACS Appl. Mater. Interfaces.

[12] Banks W.A., Sharma P., Bullock K.M., Hansen K.M., Ludwig N., Whiteside T.L. (2020). Transport of extracellular vesicles across the blood-brain barrier: Brain pharmacokinetics and effects of inflammation. Int. J. Mol. Sci..

[13] Rehman F.U., Liu Y., Zheng M., Shi B. (2022). Exosomes based strategies for brain drug delivery. Biomaterials.

[14] Ragusa M., Barbagallo D., Purrello M. (2015). Exosomes: Nanoshuttles to the future of BioMedicine. Cell Cycle.

[15] Ludwig N., Whiteside T.L., Reichert T.E. (2019). Challenges in exosome isolation and analysis in health and disease. Int. J. Mol. Sci..

[16] Lobb R.J., Becker M., Wen Wen S., Wong C.S., Wiegmans A.P., Leimgruber A., Möller A. (2015). Optimized exosome isolation protocol for cell culture supernatant and human plasma. J. Extracell. Vesicles.

[17] Chen P., Ruan A., Zhou J., Huang L., Zhang X., Ma Y., Wang Q. (2020). Extraction and identification of synovial tissue-derived exosomes by different separation techniques. J. Orthop. Surg. Res..

[18] Rekker K., Saare M., Roost A.M., Kubo A.-L., Zarovni N., Chiesi A., Salumets A., Peters M. (2014). Comparison of serum exosome isolation methods for microRNA profiling. Clin. Biochem..

[19] Faderl S., Talpaz M., Estrov Z., O’Brien S., Kurzrock R., Kantarjian H.M. (1999). The biology of chronic myeloid leukemia. N. Engl. J. Med..

[20] Rowley J.D. (1973). A new consistent chromosomal abnormality in chronic myelogenous leukaemia identified by quinacrine fluorescence and Giemsa staining. Nature.

[21] Druker B.J., Tamura S., Buchdunger E., Ohno S., Segal G.M., Fanning S., Zimmermann J., Lydon N.B. (1996). Effects of a selective inhibitor of the Abl tyrosine kinase on the growth of Bcr–Abl positive cells. Nat. Med..

[22] DeAngelo D.J., Jabbour E., Advani A. (2020). Recent advances in managing acute lymphoblastic leukemia. Am. Soc. Clin. Oncol. Educ. Book.

[23] White D.L., Dang P., Engler J., Frede A., Zrim S., Osborn M., Saunders V.A., Manley P.W., Hughes T.P. (2010). Functional activity of the OCT-1 protein is predictive of long-term outcome in patients with chronic-phase chronic myeloid leukemia treated with imatinib. J. Clin. Oncol..

[24] Tauro B.J., Greening D.W., Mathias R.A., Ji H., Mathivanan S., Scott A.M., Simpson R.J. (2012). Comparison of ultracentrifugation, density gradient separation, and immunoaffinity capture methods for isolating human colon cancer cell line LIM1863-derived exosomes. Methods.

[25] Charoenviriyakul C., Takahashi Y., Nishikawa M., Takakura Y. (2018). Preservation of exosomes at room temperature using lyophilization. Int. J. Pharm..

[26] Kim K.I., Cho B.S., Yi Y.W. (2022). Method for Lyophilizing Exosome. Google Patents. https://patentscope.wipo.int/search/en/detail.jsf?docId=WO2020027466.

[27] Arora M. (2013). Cell culture media: A review. Mater. Methods.

[28] Gardiner C., Vizio D.D., Sahoo S., Théry C., Witwer K.W., Wauben M., Hill A.F. (2016). Techniques used for the isolation and characterization of extracellular vesicles: Results of a worldwide survey. J. Extracell. Vesicles.

[29] Ding M., Wang C., Lu X., Zhang C., Zhou Z., Chen X., Zhang C.-Y., Zen K., Zhang C. (2018). Comparison of commercial exosome isolation kits for circulating exosomal microRNA profiling. Anal. Bioanal. Chem..

[30] Tang Y.-T., Huang Y.-Y., Zheng L., Qin S.-H., Xu X.-P., An T.-X., Xu Y., Wu Y.-S., Hu X.-M., Ping B.-H. (2017). Comparison of isolation methods of exosomes and exosomal RNA from cell culture medium and serum. Int. J. Mol. Med..

[31] Macías M., Rebmann V., Mateos B., Varo N., Perez-Gracia J.L., Alegre E., González Á. (2019). Comparison of six commercial serum exosome isolation methods suitable for clinical laboratories. Effect in cytokine analysis. Clin. Chem. Lab. Med. (CCLM).

[32] Gurung S., Perocheau D., Touramanidou L., Baruteau J. (2021). The exosome journey: From biogenesis to uptake and intracellular signalling. Cell Commun. Signal..

[33] Qambrani A. (2019). Recent progress in neurodegenerative diseases via exosomal therapy. Biomed. Lett..

[34] Andreu Z., Yáñez-Mó M. (2014). Tetraspanins in extracellular vesicle formation and function. Front. Immunol..

[35] Lee S.-S., Won J.-H., Lim G.J., Han J., Lee J.Y., Cho K.-O., Bae Y.-K. (2019). A novel population of extracellular vesicles smaller than exosomes promotes cell proliferation. Cell Commun. Signal..

[36] Saludas L., Garbayo E., Ruiz-Villalba A., Hernández S., Vader P., Prósper F., Blanco-Prieto M.J. (2022). Isolation methods of large and small extracellular vesicles derived from cardiovascular progenitors: A comparative study. Eur. J. Pharm. Biopharm..

[37] Wei H., Chen J., Wang S., Fu F., Zhu X., Wu C., Liu Z., Zhong G., Lin J. (2019). A nanodrug consisting of doxorubicin and exosome derived from mesenchymal stem cells for osteosarcoma treatment in vitro. Int. J. Nanomed..

[38] Rehman F.U., Liu Y., Yang Q., Yang H., Liu R., Zhang D., Muhammad P., Liu Y., Hanif S., Ismail M. (2022). Heme Oxygenase-1 targeting exosomes for temozolomide resistant glioblastoma synergistic therapy. J. Control. Release.

[39] Liang Y., Duan L., Lu J., Xia J. (2021). Engineering exosomes for targeted drug delivery. Theranostics.

[40] Zhang G., Huang X., Xiu H., Sun Y., Chen J., Cheng G., Song Z., Peng Y., Shen Y., Wang J. (2020). Extracellular vesicles: Natural liver-accumulating drug delivery vehicles for the treatment of liver diseases. J. Extracell. Vesicles.

[41] Haque S., Vaiselbuh S.R. (2021). Cd19 chimeric antigen receptor-exosome targets cd19 positive b-lineage acute lymphocytic leukemia and induces cytotoxicity. Cancers.

[42] Huang F., Wan J., Hao S., Deng X., Chen L., Ma L. (2017). TGF-β1-silenced leukemia cell-derived exosomes target dendritic cells to induce potent anti-leukemic immunity in a mouse model. Cancer Immunol. Immunother..

[43] Mian A., Rafiei A., Haberbosch I., Zeifman A., Titov I., Stroylov V., Metodieva A., Stroganov O., Novikov F., Brill B. (2015). PF-114, a potent and selective inhibitor of native and mutated BCR/ABL is active against Philadelphia chromosome-positive (Ph+) leukemias harboring the T315I mutation. Leukemia.

[44] Soekmadji C., Li B., Huang Y., Wang H., An T., Liu C., Pan W., Chen J., Cheung L., Falcon-Perez J.M. (2020). The Future of Extracellular Vesicles as Theranostics–An ISEV Meeting Report.

[45] Hosseinzadeh A., Merikhian P., Naseri N., Eisavand M.R., Farahmand L. (2022). MUC1 is a potential target to overcome trastuzumab resistance in breast cancer therapy. Cancer Cell Int..

[46] Li R., An Y., Jin T., Zhang F., He P. (2021). Detection of MUC1 protein on tumor cells and their derived exosomes for breast cancer surveillance with an electrochemiluminescence aptasensor. J. Electroanal. Chem..

[47] Zhang J., Shi J., Liu W., Zhang K., Zhao H., Zhang H., Zhang Z. (2018). A simple, specific and “on-off” type MUC1 fluorescence aptasensor based on exosomes for detection of breast cancer. Sens. Actuators B Chem..

[48] Mian A.A., Oancea C., Zhao Z., Ottmann O., Ruthardt M. (2009). Oligomerization inhibition, combined with allosteric inhibition, abrogates the transformation potential of T315I-positive BCR/ABL. Leukemia.

[49] Manniatis T., Fritsch E., Sambrock J. (1989). Molecular Cloning: A Laboratory Manual.

