# Supplementary material for: Lyophilization Based Isolation of Exosomes
Source: Int J Mol Sci. 2023 Jun 22;24(13):10477. doi: 10.3390/ijms241310477 (PMC10341925; doi:10.3390/ijms241310477)
Supplement: Supplementary file 1 [file ijms-24-10477-s001.zip › ijms-2079536-supplementary.pdf]

Supplementary data

Lyophilization Based Isolation of Exosomes

Rida e Maria Qazi [1,†], Zahra Sajid [1,†], Chunqiu Zhao [2], Irfan Hussain [1], Fizza Iftikhar [1], Muhammad Jameel [1], Fawad Ur Rehman [1,*] and Afsar Ali Mian [1,*]

[1] Centre for Regenerative Medicine and Stem Cells Research, The Aga Khan University, 1st Flour, Juma Building, Stadium Road, Karachi 74800, Sindh, Pakistan
[2] State Key Lab of Bioelectronics, Southeast University, Sipailou 2, Nanjing 210096, China
* Correspondence: rehman.fawad@aku.edu (F.U.R.); afsar.mian@aku.edu (A.A.M.)
† These authors contributed equally to this work.

Supporting Figures:

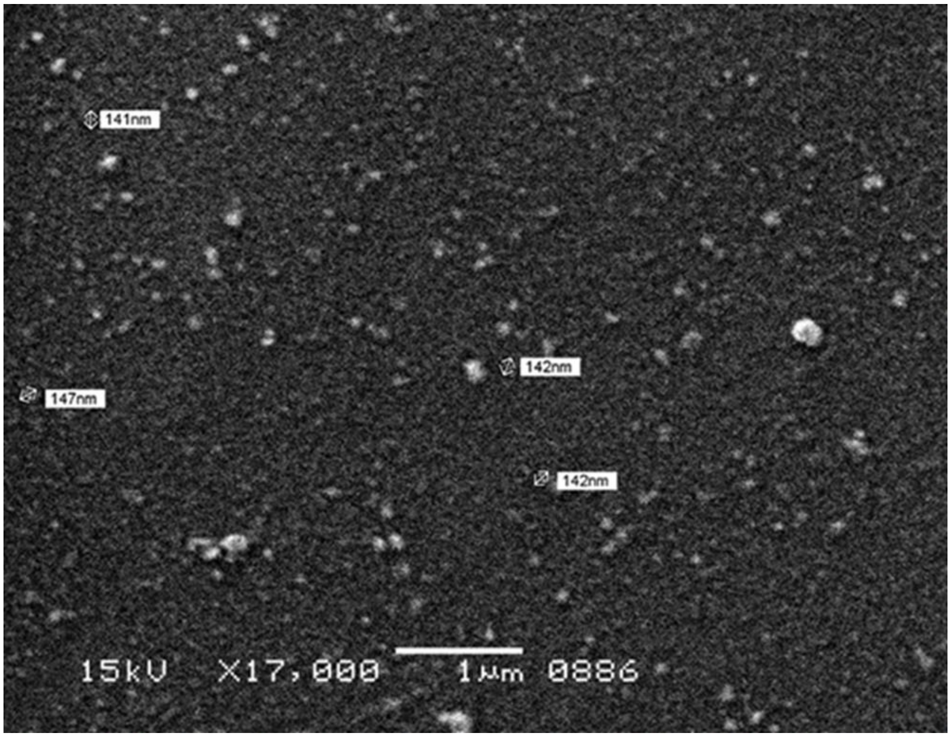

Figure S1. Scanning electron micrographs of exosomes isolated from Ba/F3-P185 cells.

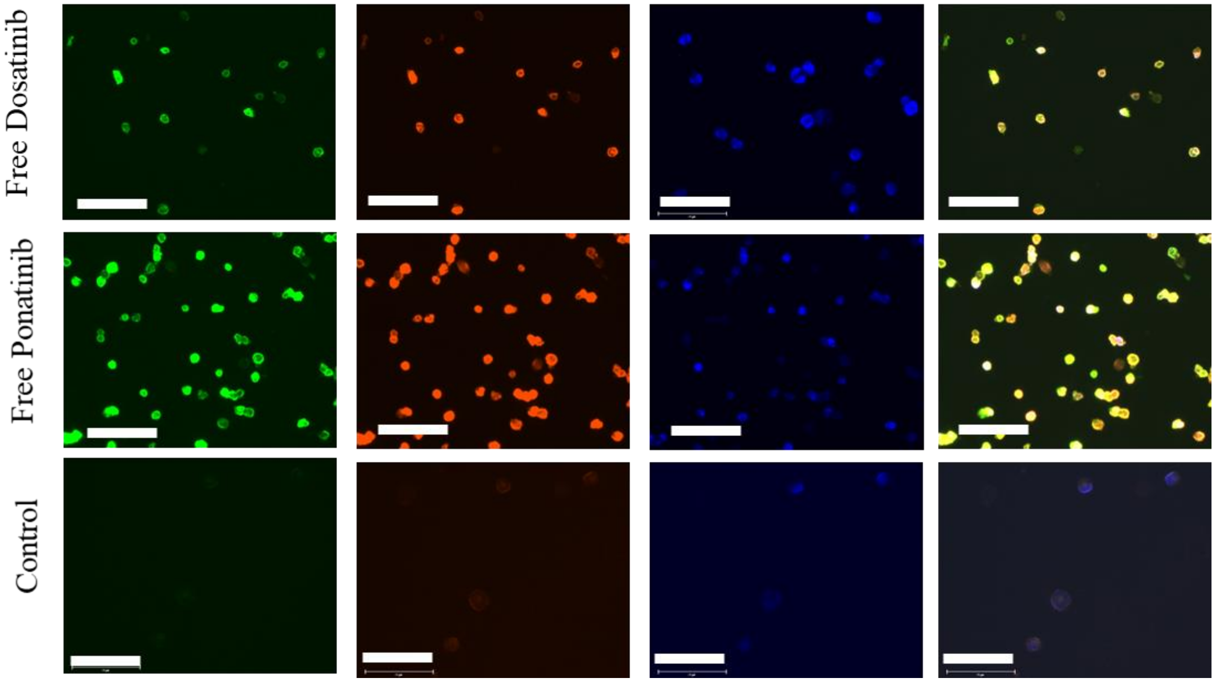

Figure S2. K562 controls for apoptosis effect after various free tyrosine kinase inhibitors.

Effect under a fluorescence microscope. The scale bar is 75 µm.

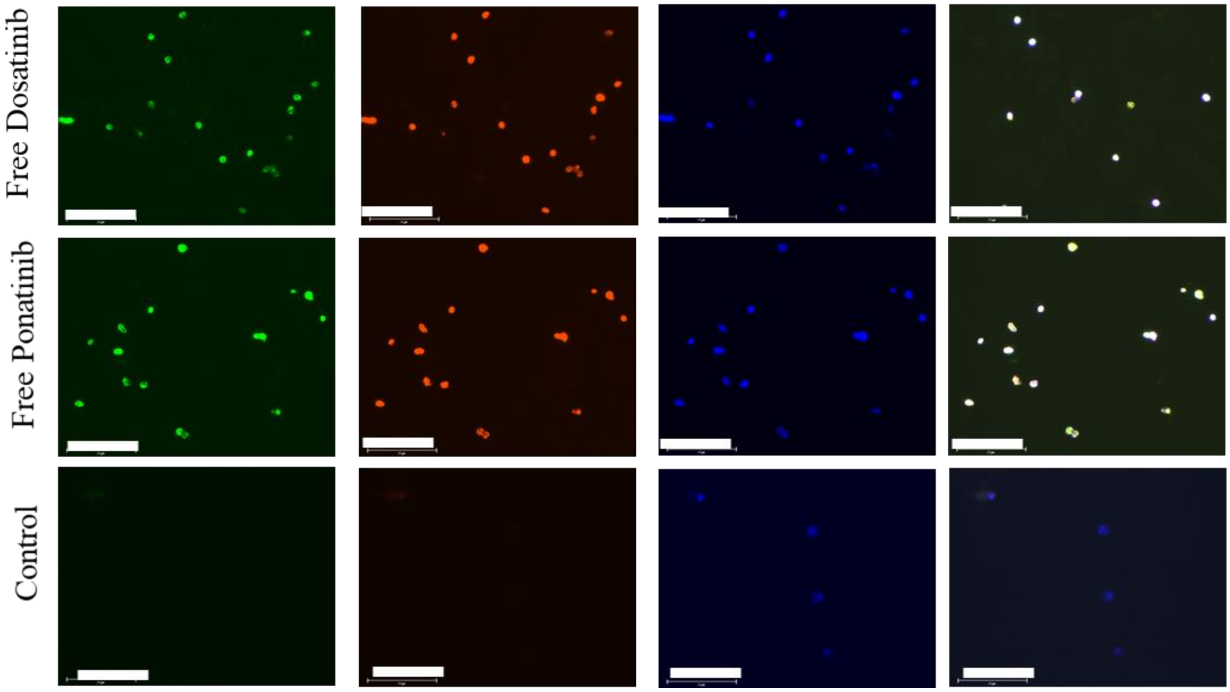

Figure S3. Ba/F3-P185 controls for apoptosis effect after various free tyrosine kinase inhibitors. Effect under a fluorescence microscope. The scale bar is 75 μm.

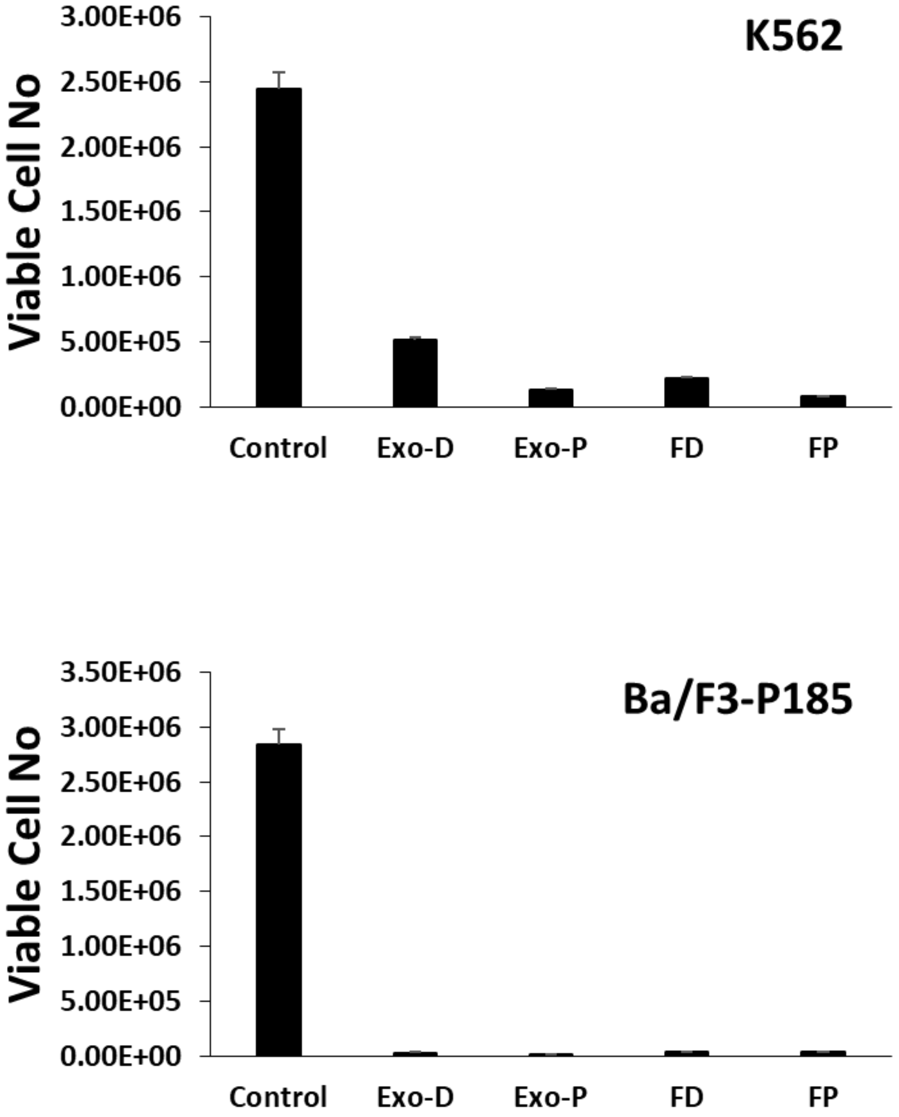

Figure S4. Viable cell count after treatment of K562 and Ba/F3-P185 cells with drug-loaded exosomes. Exo-P refers to exosomes with ponatinib, Exo-D refers to exosomes with ponatinib, whereas FP and FD represent free ponatinib and free dasatinib, respectively.

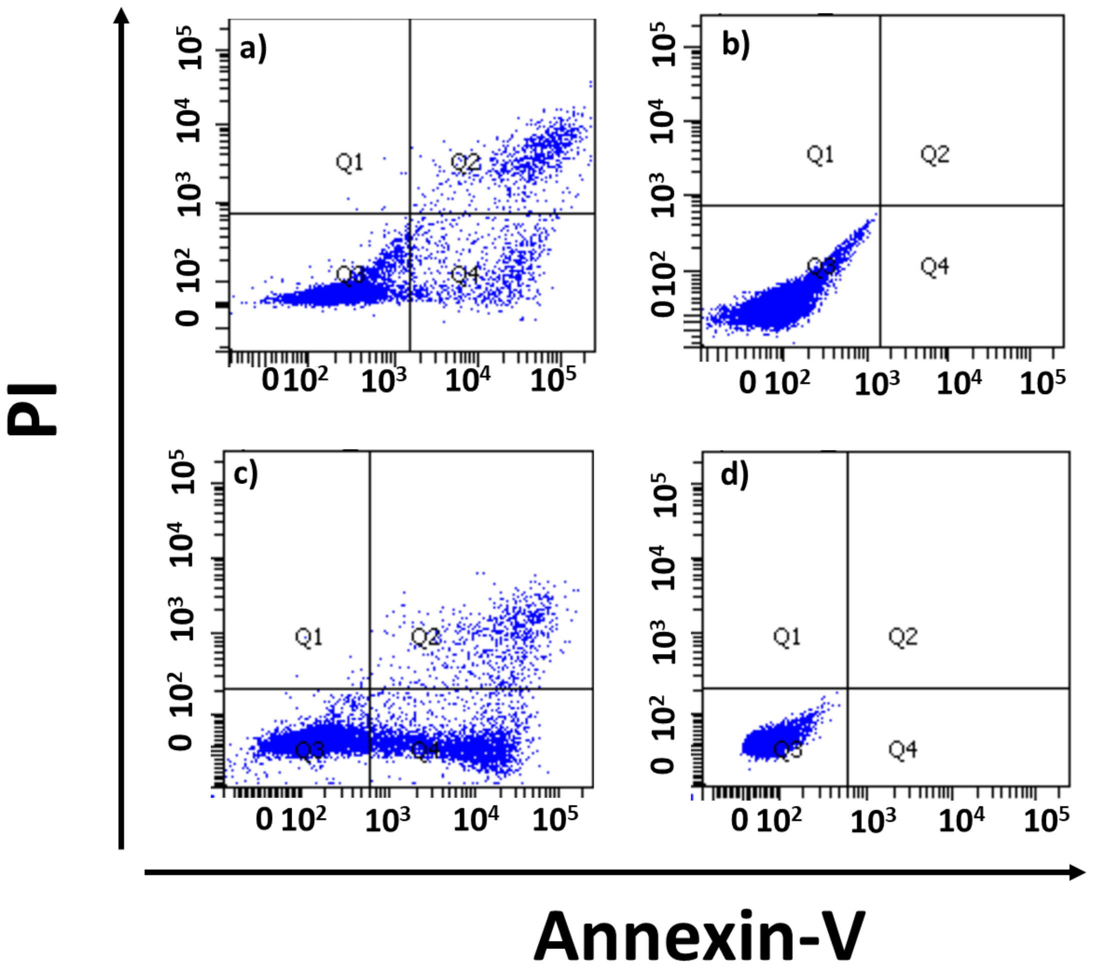

Figure S5. Flow cytometry data of Annexin V- PI stained and unstained controls of treated K562 (a, b) and Ba/F3-P185 (c, d) cells, respectively.

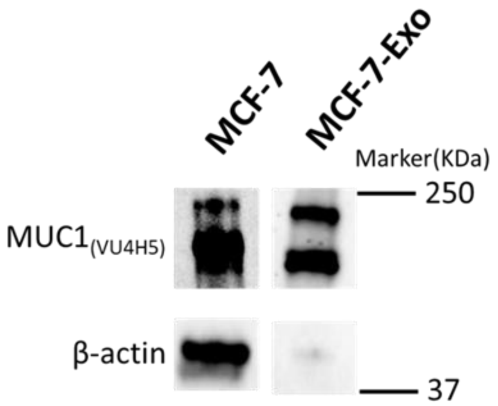

Figure S6. Western blots of MUC1 Protein expressed on the MCF7 cells and their exosomes isolated via the lyophilization method. β-actin was run as a control.
